# Current News

**Published:** 1903-04

**Authors:** 


### Current News.

### AMERICAN MEDICAL ASSOCIATION, SECTION ON STOMATOLOGY.

THE following programme will be given at the meeting of the American Medical Association, to be held in New Orleans, May 5 to 8, 1903:

1. Chairman's Address, M. L. Rhein, New York City.
2. Symposium on the Dental Pulp:
   (*a*) "Notes on Pulp Technique," Martha Anderson, Moline, Ill.
   (*b*) "Minute Structures of the Dental Pulp," Vida A. Latham, Chicago.
   (*c*) "The Vasomotor System," Eugene S. Talbot, Chicago.
   (*d*) "The Dental Pulp viewed without the Microscope," T. E. Constant, Scarborough, York, England.
   (*e*) Subject to be announced, J. Choquet, Paris, France.
   (*f*) "The Development of Hard Tissue in the Pulp," D. E. Causch, Brighton, England.
   (*g*) "Some Histological Facts Contradictory to the Theory of Odontoblasts," Michael Morgenstein, Strassburg, Germany.
   (*h*) "Pulp Hypertrophy of the Teeth," Oskär Römer, Strassburg, Germany.
   (*i*) "Tolerance of Tissues for Foreign Bodies with Special Reference to the Pulp and Peridental Membrane," M. H. Fletcher, Cincinnati, Ohio.
3. "Professional Responsibilities," A. E. Baldwin, Chicago.
4. "The Dental Symptoms of Diabetes Mellitus," Hermann Prinz, St. Louis, Mo.
5. "Germicides," A. H. Peck, Chicago.
6. Subject to be announced, G. V. I. Brown, Milwaukee, Wis.

7. " Is the Accomplishment of Reasonable Ideals in Dental Education near at Hand?" Chas. Chittenden, Madison, Wis.

8. " The Influence of Local and General Disturbances upon the Oral Secretions," Geo. F. Eames, Boston, Mass.

9. " The Dentist in the United States Navy," Richard Grady, Baltimore, Md.

10. " Methods of controlling Hemorrhage of the Oral Cavity," H. E. Belden, New Orleans, La.

11. " Empyema of the Maxillary Sinus," O. N. Heise, Cincinnati, Ohio.

12. " Medical and Dental Libraries," Alice M. Steeves, Boston.

13. " Orthodontic Facial Orthopædia," W. E. Walker, New Orleans, La.

M. L. Rhein, New York City,
*Chairman.*
Eugene S. Talbot, Chicago,
*Secretary.*

---

### AMERICAN DENTAL SOCIETY OF EUROPE.

As arranged, the Executive Committee met in Paris on December 20, Dr. Aguilar, the chairman of the Local Committee, and secretary of Section (XII.) on Odontology, of the International Medical Congress, met with them.

It was decided to accept the invitation extended to this Society and to join the Congress *en bloc,* and thus merge our meeting into that of the Section on Odontology, to be held at Madrid, Spain, April 23 to 30, 1903. The official notes have now been exchanged, and we are assured of a most pleasant season of work and pleasure while in Madrid.

At the suggestion of the American Dental Society of Europe, it was decided to have all papers in the same language read consecutively at a specified time, thus retaining the proper interest due to them and conserving the time of the members. The discussions will follow on similar lines.

A number of papers and clinics have already been promised.

It is imperative that notice be given Dr. Aguilar, sending application for membership to the Congress, together with one pound sterling, or its equivalent, and instruct him to engage hotel accommodation, which cannot be obtained unless reserved without delay.

Members of the Congress travelling on the Continent obtain a reduction of fifty per cent. in their travelling expenses and a similar reduction is expected to be granted by the English railways. Friends and families of members can obtain the same concessions upon payment of about ten shillings each in addition, application for which must be made by members joining the Congress.

We wish to repeat that the Local Committee and the Spanish dental societies are doing everything in their power to insure our comfort and pleasure.

Join the Congress.

Engage your hotel accommodation.

As the other societies are working very hard to present interesting papers, it is most essential that our full strength be shown, not only in numbers, but also in papers and clinics. The friendly rivalry of this nature should be a stimulus to our best efforts.

L. J. Mitchell,
*Honorable Secretary.*

---

### KENTUCKY STATE DENTAL ASSOCIATION.

The Kentucky State Dental Association will hold its annual meeting at Bowling Green, May 19, 20, and 21, 1903.

C. R. Shacklette,
*Secretary.*

---

### NEBRASKA STATE DENTAL SOCIETY.

The Nebraska State Dental Meeting will be held in Lincoln, Nebraska, May 19 to 22, 1903, inclusive.

H. R. Hatfield.

---

### MASSACHUSETTS DENTAL SOCIETY.

The thirty-ninth annual meeting of the Massachusetts Dental Society will be held in Mechanics' Building, Boston, Wednesday and Thursday, June 3 and 4, 1903.

Edgar O. Kinsman,

*Secretary.*

Cambridge, Mass.

